# Hypoxia: An Unusual Cause with Specific Treatment

**DOI:** 10.1155/2015/956341

**Published:** 2015-02-04

**Authors:** John P. Berger, Ganesh Raveendran, David H. Ingbar, Maneesh Bhargava

**Affiliations:** Division of Pulmonary, Allergy, Critical Care and Sleep Medicine and Division of Cardiology, Department of Medicine, University of Minnesota, MN 55455, USA

## Abstract

Hypoxia is a well-recognized consequence of venous admixture resulting from right to left intracardiac shunting. Right to left shunting is usually associated with high pulmonary artery pressure or alteration in the direction of blood flow due to an anatomical abnormality of the thorax. Surgical or percutaneous closure remains controversial; however it is performed frequently for patients presenting with clinical sequela presumed to be resulting from paradoxical embolization secondary to right to left shunting. We report two patients with hypoxia and dyspnea due to right to left shunting through a patent foramen ovale (PFO) and venous admixture in the absence of elevated pulmonary artery pressures or other predisposing conditions like pneumonectomy or diaphragmatic weakness. Percutaneous closures of the PFOs with the self-centering Amplatzer device resulted in resolution of hypoxia and symptoms related to it.

## 1. Introduction

Hypoxia due to intra-atrial communication and right to left shunting of blood is not uncommon and is associated with high pulmonary artery pressures. Less commonly, right to left shunting can occur in normal pulmonary artery pressures in the presence of structural deformities of the thorax [1–4]. We describe two cases of hypoxia and dyspnea due to right to left shunting through a patent foramen ovale (PFO) in the absence of any other predisposing thoracic cage abnormalities. These patients were treated with percutaneous closure with the Amplatzer septal occluder device with resolution of hypoxia and dyspnea.

## 2. Case Reports

### 2.1. Case 1

A 78-year-old female who was a lifelong nonsmoker with a history of multiple medical problems, including TIA, was transferred to our facility with recent seizures and weakness. Her MRI demonstrated acute left sided CNS ischemic changes, consistent with acute ischemic infarction. With supportive care she had resolution of the neurological symptoms but she remained hypoxic. An arterial blood gas obtained on 10 liters/min of supplemental oxygen showed a PaO_2_ of 36, PaCO_2_ of 37, and pH of 7.47 with oxygen saturations of 70%. Upon review of her history, this hypoxia was likely long standing as the patient's family reported her having a feeling of “not getting enough air” for the prior few months. Her family also reported that the patient used a family members' pulse oximeter at home and her “blood oxygen” was in the 78% to 88% range. A CT angiogram of the chest did not reveal thromboembolic disease or an alternative finding that could explain her hypoxia. During the hospitalization the patient remained hypoxic in both the supine and upright positions with oxygen saturations in the 70% range. On breathing 100% oxygen the O_2_ saturation failed to improve. The patient received mechanical ventilation for persistent hypoxia.

**Figure 1:**
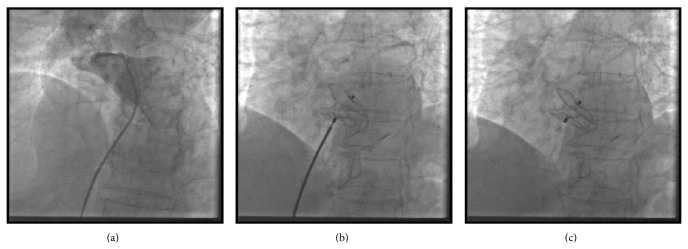
(a) 6-French multipurpose catheter passed across the PFO. A right upper lobe pulmonary venous angiogram outlines the left atrium. (b) An 18 mm Amplatzer device deployed but still attached to the release catheter. (c) Amplatzer device after release seen in stable position.

With persistent hypoxia, a history of multiple TIAs, and a stroke, the possibility of right to left shunting was considered. A transthoracic echocardiogram with agitated dextrose demonstrated a large right to left intracardiac shunt at the atrial level with a persistent Eustachian valve (see Discussion). Right heart catheterization demonstrated pulmonary artery pressures in the normal range (16/4, mean 8). The defect was balloon sized at 15 mm. An 18 mm Amplatzer septal occluder was deployed under transesophageal guidance using percutaneous catheter closure (Figures 1(a), 1(b), and 1(c)). After the procedure, the patient had rapid resolution of her hypoxia and was promptly extubated. She was discharged home on the fourth day after the procedure. At a one-month follow-up visit she reported a significant improvement in her energy and activity level and echocardiography demonstrated that the closure device was in good position. She was without hypoxia or need for oxygen supplementation. At six-month follow-up she continued to do well without needing any oxygen supplementation.

### 2.2. Case 2

A 63-year-old female was referred to the pulmonary clinic for insidious onset of dyspnea. The patient had multiple medical problems, including inflammatory bowel disease treated with prednisone varying from 10 to 60 mg/day, hypertension, hypothyroidism, peptic ulcer disease, and a distant history of lower extremity DVT. She was also using continuous positive airway pressure at night for obstructive sleep apnea with good control of her symptoms.

She had gradual onset of her dyspnea around one year prior and it had progressed to the point where she was unable to complete activities of daily living without getting short of breath. She was not hypoxic at rest with a room air oxygen saturation of 95%. Her physical examination was unremarkable. The only abnormality in her spirometry was an expiratory reserve volume of 4% predicted. The diffusion capacity was in the normal range. Arterial blood gas analysis showed a PaO_2_ of 66, PaCO_2_ of 38, and pH of 7.41. CT scanning of the chest including HRCT, inspiratory and expiratory films, and CT angiogram did not show parenchymal lung disease. Echocardiogram showed normal left atrium, right atrium, and right ventricle with a left ventricular ejection fraction of 43%. There was mild diffuse hypokinesis and a dobutamine stress echocardiogram did not show regional wall motion abnormality or valvular disease. As her symptoms were out of proportion to pulmonary abnormalities, left and right heart catheterization were done. Left heart catheterization confirmed normal coronaries and mild LV dysfunction. PA pressures were 21/7 mm Hg and PAOP was normal. Cardiac output obtained by thermodilution method was 5 liters/min. PA saturation was 69.4%.

As her symptoms worsened an echocardiogram with bubble study was done that showed early appearance of bubbles in the left atrium suggesting intracardiac shunting. Right to left shunt was confirmed on TEE. On augmentation of her heart rate by dobutamine and atropine, a significant increase in the extent of right to left shunting was seen on contrast injection. At transcatheter closure of the PFO, the size of the defect was found to be 7 mm by sizing balloon. The defect was closed with a 12 mm Amplatzer septal occluder device with no residual shunting by color Doppler at the end of the procedure. After the closure she had noticeable improvement in the exercise tolerance and O_2_ saturation. Serial echocardiograms did not reveal recurrence of the shunt or displacement of the closure device.

## 3. Discussion

In utero, the foramen ovale serves as a pathway for physiologic right to left shunting. With establishment of the pulmonary circulation at birth, there is functional and subsequent anatomic closure. PFOs can be detected in up to 30 percent of the adult population in an autopsy series [5] but are frequently considered to be asymptomatic. However PFOs may cause pathological events such as cerebrovascular, systemic embolization and diving accidents. Hypoxia is an infrequent sequela without a predisposing abnormality, bringing the underlying PFO to recognition. Though hypoxia resulting from right to left shunting in presence of high pulmonary artery pressures is well recognized, right to left shunting in presence of normal pulmonary artery pressures is less common. These cases are unique in etiology of the hypoxia and the treatment that resulted in significant improvement. It is likely that both cases had PFO for a longer duration than the observed hypoxia. As described in the next section, we speculate that the flow of blood returning to the right atrium could preferentially get directed towards the PFO and would be a possible mechanism for hypoxia. The preferential flow towards the PFO has been reported with anatomical changes and deformity of the right atrium due to age, impact from diaphragmatic ascent, pleural effusion, and changes in the ascending aorta.

### 3.1. Clinical Relevance of PFO

Using contrast echocardiography, a PFO can be detected in 5–20% of adults [6] and is usually asymptomatic. Use of Valsalva in conjunction with contrast can detect up to 60% of PFOs and the cough test detects 78% of PFOs found at the time of cardiac catherization. The presence of PFO in healthy adults may have no clinical relevance. However, hypoxia due to PFO results from venous admixture secondary to right to left shunting. A number of mechanisms have been proposed for the development of right to left shunting of blood in adults with PFO. The pressures on the right side of the heart can increase creating a gradient across the PFO. In addition, right to left shunting with near normal pulmonary artery pressures is being recognized especially in settings of pneumonectomy [1, 2], diaphragmatic paralysis [3], or elevation of right hemidiaphragm [4], kyphoscoliosis [3, 7], deformation of the atrial cavity by horizontalization, dilation of ascending aorta [8], and obstructive sleep apnea [9]. In these situations right to left shunting can occur even with normal pulmonary artery pressures or absence of permanent gradient between the right and the left atria due to preferential streaming of blood flow towards the PFO with or without an overdeveloped Eustachian valve (remnant of right valve of embryonic sinus venosus) which directs flow across foramen ovale [10]. However the exact mechanism of right to left shunting in presence of normal cardiac pressures remains uncertain [11]. The interatrial septum may be displaced towards the horizontal position, so that the atrial defect is placed directly in line with the blood flow from the inferior vena cava [12]. Right to left shunting can also occur in the absence of high pulmonary artery pressures in the setting of high gradient across PFO as seen in RV infarction [13, 14] or with obstruction to the right ventricular filling [15] as in tricuspid stenosis and right atrial myxoma. A systolic right atrium-left atrium gradient has been described [16, 17] and simultaneous measurement of pressures in both atria can establish the existence of a gradient. Abnormal oxygenation in an upright position, “the orthodeoxia-platypnea syndrome,” or failure to correct hypoxemia with increasing FiO_2_ oxygen should heighten the suspicion of right to left shunting [18]. A high index of suspicion is necessary and the pattern of occurrence of dyspnea is noted. Measurement of arterial blood gas in upright and supine positions should be made to document orthodeoxia. Further workup should include evaluation of intracardiac or extracardiac shunting.

### 3.2. Treatment for PFO

The advent of percutaneous devices has made nonsurgical closure of PFO an attractive option, especially in patients with other comorbid conditions which render them poor candidates for surgery. A number of centers have published single and multicenter reports of experience in PFO closure. Several different devices have been used with procedure success rates of 99-100% and low complication rates [19]. Long-term durability of available devices also is excellent, with a low fifteen-year failure rate [20, 21]. At present the Amplatzer, GORE HELEX, and CardioSeal septal occluders are the three devices approved for transcatheter closure of secundum atrial septal defects in the US and have been used for closure of PFOs.

Three randomized trails [22–24] evaluating death, stroke, and TIA and one trial evaluating migraine [25] failed to demonstrate beneficial effect of closure compared to medical treatment. However, given the very low event rate, none of these trails had adequate long-term follow-up to show superiority or had adequate patient population to demonstrate the benefit. MIST and Closure-I trials also used the STARFLEX technology, which is associated with increased rate of thrombus formation at the device site at 30 days [26, 27]. History of paradoxical embolization with neurological effects is still considered as an indication for closure of PFO. The diagnosis of right to left shunting can be established by injection of contrast material in the inferior vena cava with the junction of the right atrium showing complete filling of the left atrium through a patent foramen ovale. Closure of PFO to eliminate a significant shunt may benefit the patient immediately when no other alternate medical management is available.

The Amplatzer ASD occlusion device that was used in these two cases is a self-expanding double disk prosthesis made of nitinol wire mesh that is linked together by a short connecting waist [28]. The device is screwed onto a delivery cable and is delivered through a preformed sheath sized between 6- and 12-French. The waist portion of the device serves to self-center the device during deployment. Polyester patches are sewn within the disk and central stent and occlude blood flow through the device. Septal occluder devices are available from 4 to 40 mm (waist size). Cribriform device has very narrow link between the right and left atrial discs, preferentially used for PFO closure with very long tubular connection. Cribriform device is available in 18, 25, and 35 mm sizes (atrial disc size). The ideal ASD for percutaneous closure should have firm and adequately sized margins to the mitral valve, venae cavae, and the base of the aorta. PFO closures are mostly performed using the cribriform device under conscious sedation and with using intracardiac echocardiographic guidance in the cardiac catheterization laboratory. Complications associated with transcatheter closure are device embolization or malposition, arrhythmias, heart block, pericardial effusion, iliac vein dissection, groin hematoma, transient ST segment elevation, and AV block, and TIA are extremely rare.

## 4. Conclusion

Intracardiac shunting across PFO is an uncommon cause of hypoxia requiring a high index of suspicion for diagnosis in absence of history of paradoxical embolization. Percutaneous transcatheter closure is a viable option for closure of patent foramen ovale, particularly in high surgical risk patients. Recurrence of shunt is uncommon, and long-term outcome remains unknown beyond 15 years due to the recent introduction of these devices worldwide. In the one study with long-term follow-up, there was a significant difference between medical devices and medical therapy in the end point of cerebrovascular effects, especially in TIA, suggesting that there is a benefit to PFO closure in the long term. In addition, all studies to date have used a younger population (average age less than 60 years) and have not examined adverse effects of medical therapy, that is, significant bleeding. This would suggest that closure of PFO would benefit patients compared to medical therapy. With more experience and improvement in the devices, it could be a viable treatment for PFO in carefully selected patients who have physiologic consequences form the intracardiac shunting.
